# Novel advances in cardiac rehabilitation: Position paper from the Working Group on Preventive Cardiology and Cardiac Rehabilitation of the Netherlands Society of Cardiology

**DOI:** 10.1007/s12471-021-01585-4

**Published:** 2021-06-10

**Authors:** T. Vromen, R. W. M. Brouwers, H. T. Jorstad, R. A. Kraaijenhagen, R. F. Spee, M. E. Wittekoek, M. J. Cramer, J. M. C. van Hal, L. Hofstra, P. M. J. C. Kuijpers, E. C. de Melker, S. F. Rodrigo, M. Sunamura, N. H. M. K. Uszko-Lencer, H. M. Kemps

**Affiliations:** 1 Department of Cardiology, Maastricht University Medical Centre, Maastricht, The Netherlands; 2 Department of Cardiology, Máxima Medical Centre, Veldhoven, The Netherlands; 3 Department of Cardiology, Amsterdam University Medical Centre, University of Amsterdam, Amsterdam, The Netherlands; 4 CardioVitaal Cardiac Rehabilitation, Amsterdam, The Netherlands; 5 HeartLife Klinieken, Utrecht, The Netherlands; 6 Department of Cardiology, University Medical Centre Utrecht, Utrecht, The Netherlands; 7 Department of Cardiology, Slingeland Hospital, Doetinchem, The Netherlands; 8 Cardiology Centres Netherlands, Utrecht, The Netherlands; 9 Department of Cardiology, Maastricht University Medical Centre, Maastricht, The Netherlands; 10 Department of Cardiology, Onze Lieve Vrouwe Gasthuis, Amsterdam, The Netherlands; 11 Basalt Rehabilitation, Leiden, The Netherlands; 12 Capri Cardiac Rehabilitation, Rotterdam, The Netherlands; 13 Department of Industrial Design, Eindhoven University of Technology, Eindhoven, The Netherlands

**Keywords:** Cardiac rehabilitation, Telerehabilitation, Secondary prevention, Transmural care

## Abstract

Cardiac rehabilitation (CR) has evolved as an important part of the treatment of patients with cardiovascular disease. However, to date, its full potential is fairly underutilised. This review discusses new developments in CR aimed at improving participation rates and long-term effectiveness in the general cardiac population. It consecutively highlights new or challenging target groups, new delivery modes and new care pathways for CR programmes. These new or challenging target groups include patients with atrial fibrillation, obesity and cardiovascular disease, chronic coronary syndromes, (advanced) chronic heart failure with or without intracardiac devices, women and frail elderly patients. Also, the current evidence regarding cardiac telerehabilitation and loyalty programmes is discussed as new delivery modes for CR. Finally, this paper discusses novel care pathways with the integration of CR in residual risk management and transmural care pathways. These new developments can help to make optimal use of the benefits of CR. Therefore we should seize the opportunities to reshape current CR programmes, broaden their applicability and incorporate them into or combine them with other cardiovascular care programmes/pathways.

## Background and aims

Over the past few decades, cardiac rehabilitation (CR) has become an important part of the management of patients with cardiovascular diseases (CVD). Given improvements in the treatment of acute CVD, the role of CR in the modern era has changed from interventions targeted at physical recovery in the 1990s to comprehensive interventions also focussing on psychosocial aspects, lifestyle, risk factors and work resumption. Accordingly, the RAMIT trial, evaluating a low-dose CR programme, mainly focussing on short-term improvement of exercise capacity, failed to show positive effects in the modern era [1], whereas large, real-life cohort studies evaluating comprehensive multimodal programmes have demonstrated that CR is associated with a substantial survival benefit [2–4]. Furthermore, recent meta-analyses showed that multimodal CR is associated with a reduction in cardiovascular mortality and hospitalisation [5, 6]. Therefore, CR is strongly recommended in current guidelines for patients with coronary artery disease (CAD) [7]. In addition, the body of evidence for the effectiveness of CR in other patient groups is growing [8, 9].

Despite the proven effectiveness of CR, implementation remains unsatisfactory and low participation rates are a major problem. Both in the Netherlands [10] and other European countries [11], over 50% of eligible patients do not receive CR. In addition to financial barriers for upscaling CR capacity, organisational and patient-related factors can also influence participation. An important reason for non-participation is that conventional CR programmes typically are group-based and, as such, not well tailored to individual needs and preferences [12]. Logistic issues such as transportation and working obligations also form important barriers, resulting in lower participation rates [13]. A central challenge in both secondary prevention of CVD and CR in particular is the poor long-term adherence to a healthy lifestyle and the presence of considerable residual cardiovascular risk. Data from the EUROASPIRE-IV study show that risk factor control and lifestyle behaviours in CVD patients are still far from optimal [14]. Therefore, there is a need for new strategies in CR. These include, for example, more personalised CR delivery modes, preferably in the patients’ home environment. To improve (long-term) adherence, programmes should be made more appealing for a broad population and, ideally, patients should continuously be stimulated to maintain lifestyle changes, even after finishing CR. The aim of the current paper is (a) to outline the broadened scope of CR, highlighting novel and challenging target groups and (b) to discuss new developments in CR aimed at improving participation rates and long-term effectiveness in the general cardiac population by highlighting novel delivery modes and care pathways.

## Methods

This article presents a position statement from the Working Group on Preventive Cardiology and Cardiac Rehabilitation of the Netherlands Society of Cardiology. A core author group (R.B., H.J., R.S., R.K., H.K. and T.V.) was composed of members of this working group, highly experienced in the field of CR. The other members of the working group were involved as reviewers of the drafted manuscript. The core author group organised digital brainstorming sessions to determine (a) which target groups should be highlighted in this paper and (b) which new developments in Dutch CR practice should be discussed in the novel delivery modes and novel care pathways sections. This selection was based on expert opinion and a consensus meeting within the core author group and presented to the document reviewers. A group of patients was identified as ‘novel’ when there was no absolute indication for CR in the current Dutch guideline [15] but for which recent research has shown beneficial effects of CR programmes. Patient groups were identified as ‘challenging’ when recent research indicated low participation rates, or when specific tailoring of exercise programmes is required.

## Novel and challenging target groups

### Novel target groups

There is a growing amount of evidence showing beneficial effects of CR in groups that are not registered as having an absolute indication for CR in the latest Dutch guidelines [15]. These novel target groups include, but are not limited to, patients with atrial fibrillation (AF), chronic heart failure (CHF) and devices and different manifestations of the chronic coronary syndrome (CCS).

#### Atrial fibrillation

Recent trials clearly showed that risk factor modification and healthy lifestyles, in particular weight loss and exercise, are important in the management of AF patients ([16, 17]; Tab. 1). However, as mostly demonstrated by smaller trials with quality of evidence ranging from moderate to very low, CR for patients with AF seems to improve symptom burden, quality of life and exercise capacity ([9, 18]; Tab. 1). While awaiting larger trials with better methodology and longer follow-up, CR should at least be considered in all motivated patients with AF, especially in patients with obesity that wish to improve lifestyle-related risk factors [19].

**Table 1:** New evidence for the effects of cardiac rehabilitation in novel target groups

Study group	Therapy	Endpoints	Results	Reference
AF	RCT lifestyle therapy and RFM vs usual care	Arrythmia-free survival FU ≈ 6 years	HR 0.38 (95% CI 0.25–0.59)	Pathak et al. [16]
AF	Interventional study on dietary advice and exercise (no control group)	AF recurrence FU ≈ 4 years	> 10% vs 3–9% HR 0.56 (0.4–0.77) 3–9% vs < 3% HR 0.48 (0.33–0.63)	Pathak et al. [17]
AF	Systematic review and meta-analysis	Exercise capacity	1.6 (0.11–3.08) ↑ *V*O_2,peak_ (ml/kg per minute)	Smart et al. [9]
CR indication	Cohort study	CR uptake	Age > 70 9.0% Age > 80 3.3% CHF 3.7%	Van Engen-Verheul et al. [10]
VAD	Systematic review ECR	Exercise capacity Adverse events	– 2.2 ↑ *V*O_2,peak_ (ml/kg per minute) – *n* = 2 in 121 patients (1 NSVT, 1 syncope)	Alswyan et al. [8]
ICD	Systematic review ECR	Exercise capacity Shocks during exercise	– 2.6 (range: 2.2–3.2) ↑ *V*O_2,peak_ (ml/kg per minute) – 2.2%	Alswyan et al. [8]
CRT	Systematic review ECR	Exercise capacity	– 3.2 ↑ *V*O_2,peak_ (ml/kg per minute)	Alswyan et al. [8]
SAP	RCT exercise programme vs PCI	Event-free survival FU 1 year	88% vs 70% *p* < 0.001	Hambrecht et al. [23]
NOCAD	Interventional study CR (no control group)	Exercise capacity	Increase from 6.5 to 8.1 METS *p* < 0.001	Szot et al. [26]
NOCAD	Meta-analysis CR	Exercise capacity	– 31–36% ↑ WR_peak_ (W) – 15–26% ↑ *V*O_2,peak_ (l/min)	Kissel and Nikoletou [27]
Elderly CAD patients	Cohort study CR vs no CR	Mortality 5 years	34% RRR	Suaya et al. [29]
CAD	Cohort study	CR uptake	Women 15.5% Men 25.4%	Colbert et al. [32]
CAD and obesity	RCT high-caloric vs standard exercise programme	Weight loss (kg)	8.2 (SD ± 4) vs 3.7 (±5) *p* < 0.001	Ades et al. [36]
CAD and LRF	RCT lifestyle programme add-on to CR vs standard CR	Weight loss > 5%	27% vs 14% *p* < 0.001	Minneboo et al. [37]

*AF* atrial fibrillation, *CR* cardiac rehabilitation, *VAD* ventricular assist device, *ICD* implantable cardioverter-defibrillator, *CRT* cardiac resynchronisation therapy, *SAP* stable angina pectoris, *NOCAD* non-obstructive coronary artery disease, *CAD* coronary artery disease, *LRF* lifestyle risk factors, *RCT* randomized controlled trial, *RFM* risk factor management, *ECR* exercise based cardiac rehabilitation, *PCI* percutaneous coronary intervention, *FU* follow-up, *HR* hazard ratio, *CI* confidence interval, *VO*_*2,peak*_ peak oxygen uptake, *CHF* chronic heart failure, *NSVT* non-sustained ventricular tachycardia, *METS* metabolic equivalents, *WR*_*peak*_ peak work rate, *RRR* regular rate and rhythm, *SD* standard deviation

#### Chronic heart failure and devices

CHF is a relative indication for CR in the latest Dutch guideline, but in a newer European guideline it has a class 1a recommendation [15, 20]. However, CR uptake in this group appears very low [10]. Therefore, more patients with CHF should be offered a CR programme, personalised to patient characteristics in this very heterogeneous group. Within this group, patients with advanced heart failure and a ventricular assist device (VAD) are a new and emerging group attending CR. The limited number of trials for CR in this group demonstrate its safety and beneficial effects on exercise capacity ([8]; Tab. 1). In the Netherlands, only a few specialised rehabilitation centres have experience with CR in this patient group. Therefore, we recommend that CR for patients with a VAD should take place in experienced CR centres.

Current guidelines provide limited information on the effectiveness of CR in patients with an implantable cardioverter defibrillator (ICD) or cardiac resynchronisation therapy, while the number of patients with such devices is increasing [21]. A systematic review shows that exercise-based CR in patients with devices is safe and effective for improving exercise capacity and managing anxiety in ICD patients ([8]; Tab. 1). However, the optimal characteristics of exercise training in these patients remain to be determined. While awaiting more specific recommendations, as a rule of thumb, the maximal heart rate during exercise should be limited to 20 beats/min under the detection limit of the ICD [21].

#### Chronic coronary syndromes

The 2019 European Society of Cardiology (ESC) guidelines for CCS have drastically changed the management of CAD [7]. While earlier guidelines focused on stable or unstable disease, the new guidelines acknowledge the vast scope of clinical presentations that comprise CAD. Regardless of the actual stage of the CCS, the ESC endorses CR as a class 1A recommendation in all patients to achieve a healthy lifestyle and manage risk factors. However, CR in some CCS subgroups needs attention.

As such, there is an urgent need for well-conducted randomised trials of CR in patients with newly diagnosed stable angina pectoris (SAP)—with referral of patients to CR before an invasive strategy is pursued. A large recent trial demonstrated that an invasive approach is not superior to a conservative strategy in preventing major cardiovascular events or mortality in SAP patients. In this trial patients received basic lifestyle advice but did not follow a comprehensive CR programme [22]. Smaller trials showed impressive results using CR in SAP, even suggesting superiority compared with percutaneous coronary interventions ([23]; Tab. 1). The mechanism for this effect seems to be the stimulation of collateral growth ensuring better coronary perfusion [24]. Interestingly, in patients with intermittent claudication due to peripheral artery disease, exercise training and lifestyle therapy have already largely replaced vascular interventions as the treatment of first choice [25]. Whether CR is also superior to an invasive approach in patients with SAP needs to be investigated in larger trials.

Furthermore, approximately 70% of women and 30% of men undergoing a coronary angiogram for angina symptoms are found to have non-obstructive CAD (NOCAD). This large, heterogeneous group of patients comprises distinct vasomotor disorders such as vasospastic angina and microvascular disease, caused by endothelial dysfunction [27]. Adequate lifestyle measures promote well-being, reduce symptoms and improve cardiovascular function and exercise tolerance in patients with NOCAD ([26, 27]; Tab. 1). Therefore, CR should at least be considered for NOCAD patients in the future.

### Challenging target groups

Two subgroups of patients (i.e. elderly patients and women) were identified as challenging because of low participation rates [10]. Patients with obesity and CVD were classified as challenging because of the need for specific tailoring of rehabilitation programmes.

#### Frail and elderly patients

Elderly patients are highly underrepresented in CR [10] and older age seems to be a predictive factor for not following a CR programme ([28]; Tab. 1). However, patients aged above 65 years derive a large mortality benefit from attending CR [29] and show sustainable results in improved exercise capacity [30]. In 2016 the European Association of Preventive Cardiology published a call for action to include frail and elderly patients in CR programmes, as frailty can to a large degree be positively influenced by personalised CR programmes [31]. In the future, more frail and elderly patients should be offered participation in CR programmes.

#### Women

Women and men benefit equally from CR, but women attend CR less frequently than men both in the Netherlands and other European countries ([10, 32]; Tab. 1). In contrast, women mostly have worse cardiovascular risk factor profiles and lower exercise capacity [33]. Also, anxiety and depression are more likely to be present in women. These gender specificities need to be taken into account to tailor CR programmes in order to increase their uptake and positive effects in women [34].

#### Obesity

The majority of patients currently entering CR are overweight (80%) [35], and the rates of overweight and obesity, with associated negative health consequences, continue to rise globally. Also, 6 months after a cardiac incident, even after following in a CR programme, half of the patients are still overweight [14]. However, there is a steadily increasing body of evidence for successful weight loss in the setting of CR when a tailored programme is prescribed. This is demonstrated by successful initiatives such as dedicated CR weight-loss programmes showing more weight loss with high-caloric exercise programmes [36] or nurse-coordinated referral to commercial lifestyle programmes as a CR add-on ([37]; Tab. 1). The OPTICARE XL RCT (NTR6181) will give clinicians important information on the effects of a current CR programme in obese individuals.

## Novel delivery modes

### Cardiac telerehabilitation

Several alternative modes of CR delivery have been developed in an attempt to increase CR utilisation and optimise its long-term effects [38]. As such, cardiac telerehabilitation (CTR) has drawn attention in recent years. In CTR, parts of the CR programme are executed in a patient’s home environment, using remote communication (e.g. web or mobile applications) and wearable devices (e.g. heart rate monitors, accelerometers). Several meta-analyses and systematic reviews have shown that multidisciplinary or exercise-based CTR is safe and at least as (cost-)effective as traditional centre-based CR [39, 40]. The ESC therefore considers CTR to be a valid alternative to conventional CR [41] and, consequently, CTR is endorsed in the Dutch multidisciplinary CR guideline [42]. According to this guideline, remotely supervised exercise training may be recommended in low- to moderate-risk patients with CAD and remotely supervised psycho-educational prevention therapy may be recommended in low- to high-risk patients, regardless of the underlying cardiac diagnosis.

Future developments may increase the (long-term) effectiveness of CTR, for instance by applying e‑persuasion (e.g. gamification) and personalisation strategies, or by prolonging CTR towards a long-term lifestyle management intervention, incorporating relapse prevention and rewards to support sustainable healthy lifestyle behaviour.

### Loyalty programmes

Following the notion that people act in a reasoned fashion, many rehabilitation and lifestyle modification programmes focus on enhancing an individual’s health literacy, efficacy beliefs and the motivation to adopt a healthy lifestyle. This does not suffice, however, as strong drivers of health behaviours are often non-reasoned [43]. To achieve sustained healthy living, it is crucial that interventions empower patients to cope with both reasoned and non-reasoned processes and, if interventions address both, the individual and environmental factors that drive behaviour [43]. Furthermore, to ensure long-term uptake of behavioural interventions, they are preferably embedded in day-to-day routines and a patient’s home environment. In addition, meta-analyses show that rewarding health increases the adoption of a healthy lifestyle [44].

A public-private partnership, the BENEFIT consortium, uniting (academic) hospitals, rehabilitation centres, general practices, lifestyle entrepreneurs and patient federations, is currently creating an ecosystem integrating the necessary ingredients for sustained healthy living [45]. The BENEFIT approach connects initiatives from various private and public parties, such as online or offline coaching, lifestyle modification applications and self-monitoring devices. Healthy living is made more attractive with challenges and rewards for attending appointments with healthcare providers, adhering to evidence-based lifestyle-change programmes and coaching and self-monitoring lifestyle behaviours. These rewards can be exchanged for discounts on health-related goods and services. Patients are provided with a personal health application which can store their health-related information, connect with wearables, supports teleconsultation and provides access to digital lifestyle interventions. Within their digital environment, patients can share data with health professionals in the different care settings, enabling coaching and monitoring of patients ‘at a distance’ and ensuring the continuity of care and transmural access to lifestyle interventions. The BENEFIT approach is currently being evaluated in a stepped-wedge roll-out.

## Novel care pathways

Currently, CR and traditional cardiovascular risk management are often suboptimally integrated. Whereas CR is usually targeted at physical training, psychosocial recovery and lifestyle management only, medical treatment of traditional risk factors such as hypertension, dyslipidaemia and diabetes is performed separately. Yet, it has been shown that an integrated approach including both lifestyle therapy and medical treatment can be particularly effective [25, 46]. Also, poor alignment of CR programmes with primary care secondary prevention programmes hampers the optimal long-term effectiveness of CR. Typically, a CR programme lasts for 3 months and patients are included in primary care cardiovascular risk management programmes substantially later, often 1 year after a cardiovascular incident. This results in a treatment gap, frequently leading to discontinuing personalised long-term prevention goals set out during the CR programme and consequently to a relapse into unhealthy behaviours [14].

### Integration of CR in residual risk management

Residual risk, i.e. ‘the risk of new vascular events or progression of established vascular damage’ is an important emerging challenge in CR and secondary prevention of CVD [47]. While healthy lifestyle changes form the cornerstone of any strategy to reduce residual risk, numerous new and upcoming pharmacological treatment options are increasingly becoming available for patients with CVD. These include intensive low-density-lipoprotein-lowering strategies, new antithrombotic and anticoagulation strategies and new antidiabetic treatment options [7]. Pending data from ongoing or recently published trials, novel pharmacological residual risk treatment targets are also becoming available, such as triglyceride level, inflammation, lipoprotein A level and even obesity [48, 49]. However, there is an unmet need for a consensus as well as for decision-making tools to help clinicians identify which patients should receive which pharmacological treatment, in which sequence, to what degree or level of intensity. Patient perspectives and preferences are urgently needed when designing clinical programmes to address residual risk. Furthermore, a number of the new pharmacological strategies carry considerable costs, and cost-effectiveness should be taken into account, especially against the background of comparable effects through lifestyle changes which might also be achieved through successful CR programmes. After the initiation of new pharmacological treatments, monitoring should take place throughout, also after CR, and be clearly communicated when patients are discharged back to primary care. Additionally, education and lifestyle modifications can have an important impact on adherence to and maximising the effects of pharmacological treatment. CR has been shown to promote better medication adherence in patients after an acute coronary syndrome (ACS), and rates of adherence to pharmacological and lifestyle treatments are independently associated [50]. Therefore, integration of pharmacological residual risk management with CR and a seamless transition to long-term management by primary caregivers is essential to optimise long-term risk management.

### Transmural care pathways

Transmural care pathways can be an important facilitator for better alignment of CR programmes with primary care risk management, enabling the transfer of prevention programme components out of the CR centre at an early stage when possible. On the other hand, they facilitate the extension of duration of treatment modules inside the CR centre when indicated (e.g. in cases of high disease complexity). As an example, in non-complex CR patients the exercise programme can take place in a primary care physical therapy practice near the patients’ home environment. However, if parts of the CR programme are performed outside the CR centre, quality of care should be warranted by appropriate education of staff, monitoring of treatment results and benchmarking. In addition, the CR centre should still coordinate the multidisciplinary care of CR patients and, if necessary, primary caregivers should be given the opportunity to participate in multidisciplinary team meetings. Another requirement for the implementation of such transmural care programmes is reimbursement for exercise programmes in primary care, which should be nationally available. Finally, to ensure collaboration between primary caregivers and hospitals or CR centres, connected and synchronised ICT systems should be developed to suit both parties’ needs. An example of a transmural care pathway is the Netherlands Society of Cardiology (NVVC) Connect programme with transmural agreements and collaboration for patients after an ACS, launched in 2011. Furthermore, *Chronisch Zorgnet*—a network of specialised primary care physical therapists for the delivery of primary care exercise programmes in the Netherlands—is currently being formed. Collaborating with such a network as part of the national NVVC Connect programme may help to increase the quality of care delivered by both primary and secondary caregivers.

## Conclusion and future directions

CR has evolved as an important part of the treatment of CVD. However, to date, its full potential is fairly underutilised. Improving adherence to CR, especially in underrepresented patient groups, and providing CR for new target groups that would likely benefit from CR, could further reduce morbidity and mortality. Furthermore, telerehabilitation and loyalty programmes are promising new CR delivery modes to increase participation and to optimise the long-term effects of CR. Finally, CR and secondary prevention programmes may be improved substantially by the integration of CR in residual risk management and transmural care pathways. To benefit fully from the potential of CR, we should seize the opportunities to reshape current CR programmes, broaden their applicability and incorporate them into or combine them with other cardiovascular care programmes.
